# Challenge, integration, and change: ChatGPT and future anatomical education

**DOI:** 10.1080/10872981.2024.2304973

**Published:** 2024-01-13

**Authors:** Lige Leng

**Affiliations:** Fujian Provincial Key Laboratory of Neurodegenerative Disease and Aging Research, Institute of Neuroscience, School of Medicine, Xiamen University, Xiamen, Fujian, P.R. China

**Keywords:** ChatGPT, artificial intelligence, anatomy, medical education, educational reform

## Abstract

With the vigorous development of ChatGPT and its application in the field of education, a new era of the collaborative development of human and artificial intelligence and the symbiosis of education has come. Integrating artificial intelligence (AI) into medical education has the potential to revolutionize it. Large language models, such as ChatGPT, can be used as virtual teaching aids to provide students with individualized and immediate medical knowledge, and conduct interactive simulation learning and detection. In this paper, we discuss the application of ChatGPT in anatomy teaching and its various application levels based on our own teaching experiences, and discuss the advantages and disadvantages of ChatGPT in anatomy teaching. ChatGPT increases student engagement and strengthens students’ ability to learn independently. At the same time, ChatGPT faces many challenges and limitations in medical education. Medical educators must keep pace with the rapid changes in technology, taking into account ChatGPT’s impact on curriculum design, assessment strategies and teaching methods. Discussing the application of ChatGPT in medical education, especially anatomy teaching, is helpful to the effective integration and application of artificial intelligence tools in medical education.

## Introduction

ChatGPT is a chatbot program developed by OpenAI, which was released on 30 November 2022. ChatGPT is a natural language processing tool powered by artificial intelligence technology. Its full name is ‘Chat Generative Pre-trained Transformer (ChatGPT)’ [1]. ChatGPT was originally developed in 2018, and InstructGPT, or GPT3, came out in 2022. ChatGPT is an original based on the principle of InstructGPT. Generally speaking, it needs to handle global correlation between words and words. Therefore, the main work of ChatGPT is to realize the generality of GPT model, that is, to make GPT speak like a human and simulate the thoughts and ways of human language. In order to do this simulation well, ChatGPT also needs to go through a series of training processes to form a supervised learning model, a training reward model and a reinforcement learning fine-tuning generation model. In terms of practical application, ChatGPT covers a wide range of fields, including consulting and education, art and entertainment, IT technology, medical and health, etc.

When people are rushing to discuss the important impact of the new generation of generative artificial intelligence technology such as ChatGPT on education reform, another more prerequisite understanding problem may be overlooked by everyone, that is, what is the connotation and positioning of ChatGPT in the perspective of education? Although ChatGPT may replace simple human labor and general intelligence, this does not mean that education will cease to exist. On the contrary, it forces human beings to explore and engage in more creative labor, forces human beings to change the original way of education and learning, and puts forward higher requirements for the future development of human beings. Because in essence, human creativity is always not possessed by intelligent machines, intelligent machines can only be a recombination of language information, and people are always the creators of machines. Therefore, whether it is for teachers’ teaching and students’ learning, ChatGPT is a good assistant and a strong text analysis tool. It can use powerful information combination ability to analyze and combine existing texts to produce ‘new’ text content, but it cannot generate original ideas like human beings, because it lacks original ability. Therefore, machine intelligence only provides a convenient tool and carrier for people, and it is impossible to surpass the subject status of people. In terms of tool value, ChatGPT, like other technologies, is driving changes in the way education is conducted, such as enabling researchers to quickly access information, generate automated literature reviews, process large amounts of text data, facilitate personalized learning for students, enhance interaction and real-time feedback, and provide multilingual support. However, these ‘can’ also hide its many ‘difficulties’, such as the limitation of ChatGPT’s professional knowledge, the limitation of training data, the limitation of language understanding and reasoning ability, the lack of emotional communication, professionalism and depth, misdirection and errors, and privacy and security issues, will bring the corresponding education problems. These problems include attaching too much importance to knowledge teaching and neglecting to educate people, over-reliance on machines and lack of independent thinking, academic cheating and academic integrity problems, and hindering students’ emotional development and the formation of sound personality.

**Figure 1.**
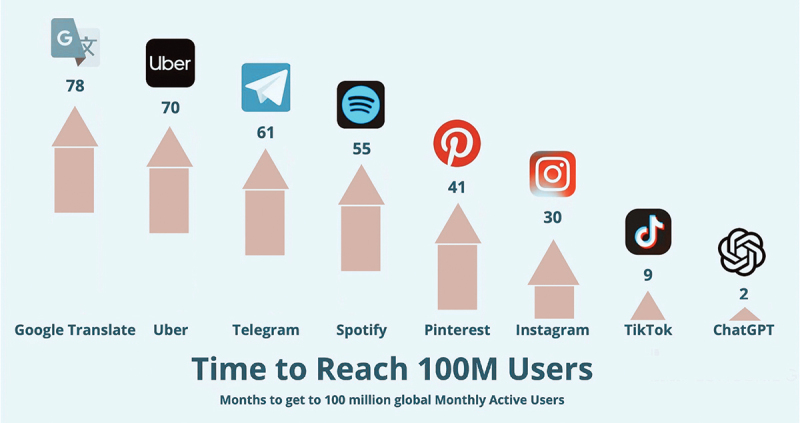
ChatGPT reached over 100 million users in just two months.

ChatGPT has both positive and negative effects on medical education [2,3]. The integration of ChatGPT with medical education is a continuous and gradual process [4,5]. First, its emergence offers exciting new opportunities for educational innovation. ChatGPT received considerable attention for its ability to generate human-like text and engage users in interactive conversations, thus gaining 100 million users within two months (Figure 1). With its natural language processing capabilities and advanced algorithms, ChatGPT can effectively automate time-intensive tasks such as summarizing and evaluating relevant medical knowledge and medical literature. ChatGPT has the potential to provide students with detailed and personalized information in medical education and can be paired with other technical software to develop interactive simulations. The application of ChatGPT in medical education has the potential to revolutionize the medical education model. By acting as a virtual teacher, ChatGPT can provide real-time and personalized feedback to students [6].

**Figure 2.**
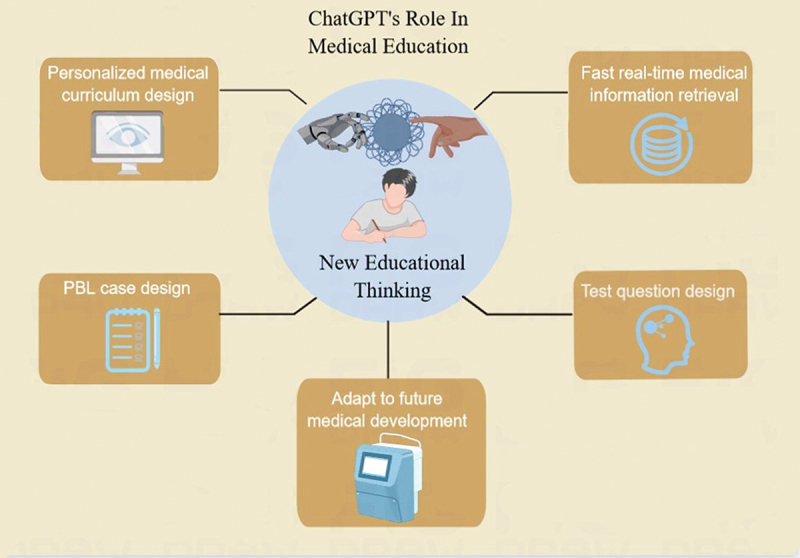
ChatGPT’s role on medical education.

**Figure 3.**
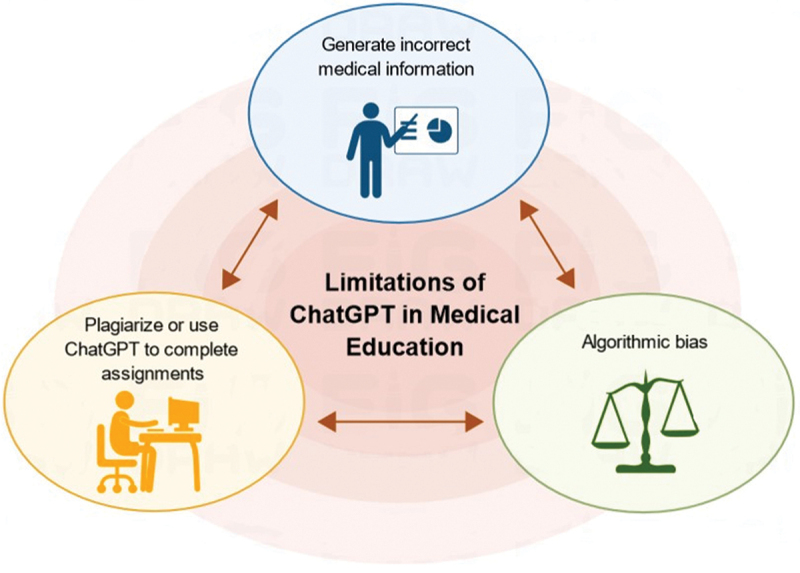
Limitations of ChatGPT on medical education.

In this paper, we analyze in detail the advantages and limitations of ChatGPT in medical education, especially in the teaching of anatomy [7] (Figures 2 and 3). In addition, we explore the impact of ChatGPT on PBL and discuss future research and development directions to maximize the potential of this technology.

## Description

### Potential application and benefits of ChatGPT in medical education

#### ChatGPT provides instant personalized medical information

ChatGPT can be used as a medical education aid, with its ability to quickly collect information on a variety of medical topics to help students quickly and real-time [8]. ChatGPT provides immediate feedback and 24-hour access to information, answer and anatomy, histology and other medical subjects related problem. Proficient use of ChatGPT by students can reduce the workload of teachers. Another benefit of using ChatGPT is its potential to provide personalized education. ChatGPT can track students’ progress and adjust their teaching style in a way that suits their level, simplifying and explaining complex concepts. For example, a first-year medical student can select ‘elementary level Interpretation [definition]’ to have a conversation with ChatGPT [9].

#### ChatGPT can provide test questions to measure the progress and effectiveness of learning

**Figure 4.**
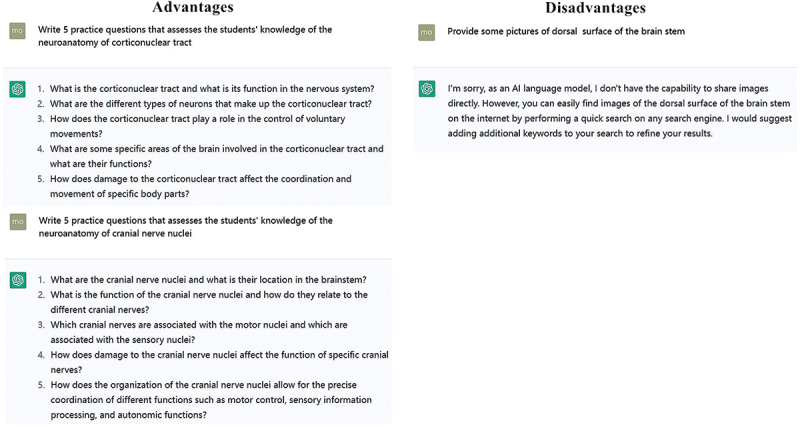
ChatGPT can easily provide test questions on request, but its ability to read, collect and generate pictures is poor.

According to the learning results of each stage, ChatGPT can provide test questions (left side of Figure 4) according to the needs of students to test the learning effect. Students can further check the gaps and make up for the learning process according to their own answers [10,11]. As can be seen in Figure 4, ChatGPT can easily provide questions on demand, examining the anatomical knowledge of the cortical and neural tracts of the brain. Not only can the questions be personalized to meet the specific situation of each student, but also can greatly reduce the workload of teachers.

When we evaluated the generating questions on anatomy, we found that ChatGPT was quite good. Even though ChatGPT may generated incorrect questions, it can be used after the inspections to make exam questions of different difficulty and can save teachers’ time [12]. Moreover, while preparing a question, it can also suggest different alternative questions [13,14].

Thus, the ChatGPT has the potential to become a more reliable and valuable tool for anatomy education and exam preparation by improving the ability to understand anatomy-related concepts and generate appropriate questions and distractors for different difficulties [15].

#### ChatGPT can write cases to assist the teaching of problem-based learning (PBL)

**Figure 5.**
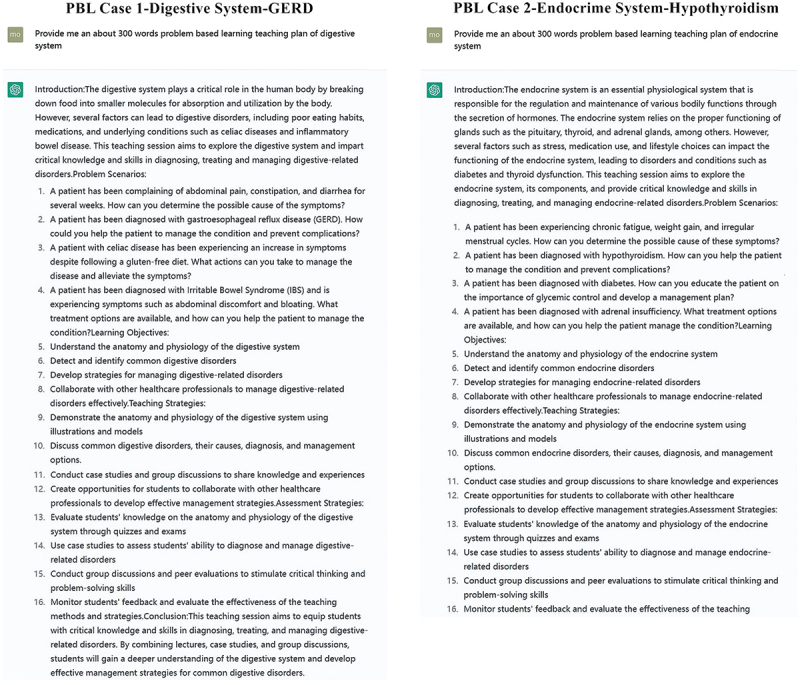
ChatGPT can easily design PBL teaching cases.

PBL teaching is a problem-oriented teaching method, which mainly adopts heuristic, self-study and interactive discussion teaching. However, anatomy is a course that freshmen learn in college, and students’ medical knowledge is almost blank before learning human anatomy course. Therefore, attention should be paid to the simple and easy to understand PBL case writing. It is difficult for students to master complex and massive anatomical knowledge solely through PBL teaching method, and it is difficult to deeply discuss clinical cases. The purpose of local anatomy experiment class is to complete the understanding and understanding of normal human body morphology and structure through practical operations such as viewing, touching and dissecting. If too much emphasis is placed on the discussion of PBL cases, it will make students attach importance to theory and despise practice, and prefer books to despise specimens. Therefore, to carry out PBL teaching correctly and fully prepared is the premise of successful teaching reform. PBL case teaching materials are comprehensive and contain many basic medical disciplines and clinical knowledge based on form. Therefore, case preparation requires that the authors should have rich knowledge of morphology and clinical medicine, certain teaching and clinical experience, careful thinking and rigorous logic, and the ability to raise high-level questions closely related to morphological teaching. For morphology teachers, it is very difficult to write high-quality PBL teaching cases independently. ChatGPT can write cases to assist PBL teaching (Figure 5). In Figure 5, we can see that ChatGPT can write relevant PBL teaching plans as required, providing PBL-related cases for digestive system and endocrine system. Teachers can refine PBL cases based on ChatGPT to make them more suitable for students’ learning.

#### ChatGPT can design the medical course schedule

ChatGPT has the potential to have a significant impact on curriculum design, assessment strategies and teaching methods in the field of medical education. In the process of our anatomy teaching exploration, we gradually develop a hybrid online-offline course, in which students learn the comprehensive content of anatomy and pathology, and through the interactive synthesis of online textbooks, exercises, cases, literature and ChatGPT. The course learning plan will be adjusted from time to time according to the students’ learning situation, so as to extend the learning time and deepen the learning depth for the poor understanding and mastering of the content in the first learning. After the total online study time reaches 45 hours, students can take an online exam.

As AI technologies continue to play an increasingly important role in medical education, educators must adapt to the changing landscape and ensure that students are equipped with the necessary digital literacy and capabilities to effectively utilize advanced technologies in future medical work and integrate them into clinical practice. To achieve this, medical educators need to think about our teaching methods and incorporate AI-based teaching methods into our curriculum. In the future of healthcare, digital tools such as online platforms [16,17], digital applications and electronic health records will be ubiquitous [18]. Ai-assisted diagnosis is also likely to be increasingly trusted. Therefore, students should acquire the ability to effectively apply digital tools to meet the needs of future medical work.

#### ChatGPT’s reference to educational thinking

With the emergence of ChatGPT and its application development in the field of education, a new era of collaborative development and educational symbiosis of human intelligence and artificial intelligence has come, and reshaping the technological influence of artificial intelligence to human education reform has become very critical. First of all, based on four dimensions, which are the basic principle of ChatGPT and its educational impact, the emergence of intelligence and its educational thinking, the ability boundary and its educational application, and the value of tools and its educational problems, we have gradually realized that the value and impact of ChatGPT’s characteristics and functions on human education. Secondly, in the process of relationship reconstruction, ChatGPT also makes education face many challenges, such as ‘how can education preserve the dignity of human beings?’, ‘how can teachers survive in desperate circumstances?’, ‘what is the most important ability and quality of students?’ and ‘how can Chinese education cultivate innovative talents?’, which have triggered our new thinking. Third, we must put forward targeted strategies from many aspects, such as strengthening the mutual transformation of high-quality data and valuable knowledge, strengthen the educational ability of teachers to cooperate with human intelligence and artificial intelligence, strengthening the cultivation of students’ all-round digital literacy, and strengthening the design of the integrated educational development model of ‘integration education’. Then, based on the new picture of human-machine integration, we find that human education and learning patterns, the historical narrative of human education, the transformation of human education and the production tools of educational research are all changing. Finally, under the influence of generative AI, the pivotal reform of future educational is quietly taking place, including the birth of ‘composite educators’, the remodeling of the underlying logic of education.

ChatGPT needs to be trained by a large amount of data over a long period of time, and it is difficult to solve problems in one step. In fact, it is the same for people, and large problems need to be decomposed into small problems to achieve the purpose of solving. ChatGPT is an application of the chain of thought, in which a large problem is broken down into smaller ones, and the machine is guided to dismantle, think, reason, and solve the problem step by step. However, it is the educational thinking of this chain of thought that helps ChatGPT achieve the natural result of ‘intelligent emergence’. This is also of reference significance for our educational thinking reform. In the usual teaching, we should also pay attention to cultivate the students’ initiative, commands through a problem-solving skills and clinical thinking.

### Limitations of ChatGPT in medical education

#### ChatGPT may generate incorrect medical information

Despite ChatGPT’s existing advantages and future potential, the biggest major challenge is to ensure the accuracy and reliability of the information provided by the AI system. Medical education requires a high degree of precision and accuracy, as even minor errors can have a significant impact on patient safety. ChatGPT is not a database, but a language model that is trained with a large amount of data. In its training process, there is the possibility of errors or omissions. If ChatGPT gives wrong answers, it is difficult for students to recognize such errors. To ensure the accuracy of ChatGPT in medical education, clear guidelines should be developed and extensively tested and inspected by institutions designated by government education authorities.

Previous study on ChatGPT question answering accuracy was found to be 72.5% accurate on anatomical knowledge. Even on vessel-related questions, which had the highest accuracy, ChatGPT did not achieve 85% accuracy, suggesting that we should be more cautious about applying chatGPT for medical education [19,20]. Besides that, ChatGPT can make fact-based changes based on human instructions. Such training that deviates from the facts can lead to false conclusions in later answers.

ChatGPT-4 cannot conduct real-time searches for articles, even if the author’s information is provided. This was highlighted in two previous articles [7,21], where the chatbot provided summaries of fake articles, which received criticism in the comments. However, if the user provides the chatbot with the full text of an article, it can then provide a summary. ChatGPT-4 emphasizes the importance of learning anatomy from various perspectives to promote a thorough understanding of the subject matter and its value.

#### Students may plagiarize or use ChatGPT to complete assignments

Students may use ChatGPT to complete assignments without further study. This will reduce students’ ability to think critically, be creative and synthesize information in the learning process. Plagiarism detection techniques such as original.ai, GPTZero, and Plagibot can be used to screen for this condition.

#### ChatGPT has poor ability to recognize and use pictures

Although ChatGPT4 has greatly improved the ability to read pictures, it is far from satisfying the normal picture understanding function. Moreover, ChatGPT has no good ability to search and generate pictures, which greatly limits the basic medical disciplines such as morphology, which have a high demand for pictures (right side of Figure 4).

#### ChatGPT’s medical education may have an algorithmic bias

One of ChatGPT’s major ethical concerns in medical education is the bias and discrimination that these models have the potential to iterate. If the data and algorithms used to train the model are biased, then the trained ChatGPT will reproduce and amplify biases, such as gender and race biases [22,23], which will adversely affect students in various subjects, including medical education.

## Discussion

ChatGPT is undergoing rapid development and has a profound impact on medical education. More and more studies have begun to explore the possibility of applying ChatGPT in medical education. It must be continuously studied and evaluated to ensure that AI systems can be better integrated and play a positive role. Therefore, the authors gradually tried to apply ChatGPT from the anatomy teaching process. In this paper, the author expounds the advantages and disadvantages of ChatGPT in anatomy teaching not only through reading literature, but also through her own experience and practice in teaching. In the future teaching work, if this technology can be organically combined with traditional teaching methods, it will certainly benefit both teachers and students. A detailed evaluation of the advantages and disadvantages of ChatGPT will help to bring its efficacy into full play, avoid its disadvantages, and provide a basis for the evaluation and supervision of the application of ChatGPT in medical education.

Faced with the iterative upgrading of artificial intelligence dialogue robots and the intervention of education, the ecology of human education has become more complex, the profession and development of teachers have been strongly impacted, and the question, challenge and danger of whether teachers will be replaced have come one after another. In the face of new technologies, new tools and even new subjects, teachers should rise to the challenge and become a reflective educator, that is, based on the understanding and reflection of education and learning, think about ‘how students learn’ in today’s great change in learning mode, think about ‘why we teach what we teach’, and become a new educator who ‘teaches for thinking and teaches for literacy’. Actively promote the transformation and upgrading of modern education and teaching, and truly liberate the limitations of students’ learning of ‘practical knowledge’.

In the process of answering specific anatomical questions, we noticed some information incorrectly provided or omitted by ChatGPT. It may not provide much information on anatomical variants, while it does highlight their clinical impact. The updated version of ChatGPT was utilized which is believed to be better than its previous versions [20]. However, the accuracy of the replies provided may vary depending on the nature of the question asked.

The development of large language models (LLMs) including ChatGPT are evolving every day. Therefore, more studies are needed to fully explore its capabilities in the future. However, these abilities will develop through deep learning. Since our main language is Chinese, both English and Chinese are used in our tests. When we tested the performance of the models in different languages, we did not observe any difference between ChatGPT. We evaluated that the dataset of these data software did not make any difference in response to the questions in different languages, which showed that the language options were working well. We also tested that the success does not increase if the questions are repeated as a result of machine learning.

Medical course and training in low- and middle-income countries (LMICs) have continuously encountered barriers and challenges [24]. Due to the large population in China, medical resources are relatively scarce and the relationship between doctors and patients is tense. However, the medical development is extremely rapid, which results in the relative shortage of medical education resources. In addition, China is a relatively traditional country, and body donations are relatively scarce. All these have caused some difficulties to the development of anatomy teaching. Challenges for medical education in LMICs can be attributed to insufficient financial and technological resources, undeveloped education models (i.e., teacher-centered approach instead of student-centered approach), and a lack of updated medical information. ChatGPT can serve as a valuable supplementary tool for medical education in resource-limited settings. ChatGPT allows medical students to ask questions about specific medical concepts and receive accurate and personalized responses to help them better structure their knowledge around each concept. This holds promising potential, especially in LMICs where traditional medical education resources may be scarce. Furthermore, ChatGPT can offer suggestions to medical educators regarding course outlines and structure, further augmenting the medical education process. It can also revolutionize medical education by simulating case reports, offering a dynamic, case-based learning approach. This is invaluable when access to real patient data is limited due to consent or confidentiality concerns. ChatGPT’s ability to generate realistic yet fictionalized patient interactions not only ensures diverse clinical exposure but also upholds ethical standards. By integrating this tool into medical curricula, students can engage with simulated cases in real-time, while fostering interactive learning and critical thinking. Leveraging on ChatGPT as a pedagogy tool may represent a new paradigm shift in blended learning. Finally, medical training and education are lifelong processes for healthcare professionals, and keeping up with the latest research, techniques, and guidelines can be challenging, especially in LMICs. Through plugins, ChatGPT can provide instant access to relevant up-to-date medical information and resources for healthcare professionals, making it easier to support their ongoing learning and development, improve their skills and knowledge, and therefore provide better care to patients. However, the computational power demanded by ChatGPT currently necessitates dedicated servers and internet connections, which poses challenges for adoption in LMICs due to infrastructure limitations and insufficient internet access or electronic devices. According to a report from the Alliance for Affordable Internet (A4AI), basic internet access is only available to half of the population in LMICs, and of those, only 10% have what is referred to as ‘meaningful connectivity’ or a decent internet connection [24]. The ongoing development of LLMs and innovative internet solutions may hold promise for mitigating these challenges in the near future.

Information technology has a revolutionary impact on education. As an important productive force affecting the development of education, it plays an important role in the reconstruction of teaching mode, education mode and education ecology. In particular, the addition of new artificial intelligence technology products represented by ChatGPT further highlights the revolutionary impact of technological iteration and upgrading on future education. And it will have a big impact on the next round of education reform. The impact mainly occurs on learners, teachers and researchers. For learners, the problem of learning motivation becomes more serious. Positive motivation can promote students’ autonomous learning and personalized learning, but negative motivation may lead to trust hazards such as cheating. For teachers, the human-computer mixed teaching environment is helpful for the design of teaching content, the optimization of teaching process, and the deepening of teaching evaluation. However, with the continuous evolution of ChatGPT, the key challenge for teachers is the problem of ‘what to teach’. As it is difficult for learners and teachers to ignore the self-limitation of technological development, researchers need to study the new opportunities brought by new education and new laws, especially in the new ‘teacher-ChatGPT-student’ ternary education scene and environment, to promote the rapid development of intelligent education and intelligent pedagogy, which also leads to an important change in the paradigm of education research.

## Data Availability

All data needed to evaluate the conclusions in the paper are present in the paper.
